# The Carboxy-Terminal Domain of Erb1 Is a Seven-Bladed ß-Propeller that Binds RNA

**DOI:** 10.1371/journal.pone.0123463

**Published:** 2015-04-16

**Authors:** Wegrecki Marcin, Jose Luis Neira, Jeronimo Bravo

**Affiliations:** 1 Instituto de Biomedicina de Valencia, Consejo Superior de Investigaciones Científicas, c/ Jaime Roig 11, 46010 Valencia, Spain; 2 Instituto de Biología Molecular y Celular, Universidad Miguel Hernández, Avda. del Ferrocarril s/n, 03202 Elche (Alicante), Spain; 3 Instituto de Biocomputación y Física de los Sistemas Complejos (BIFI), 50009 Zaragoza, Spain; Molecular Biology Institute of Barcelona, CSIC, SPAIN

## Abstract

Erb1 (Eukaryotic Ribosome Biogenesis 1) protein is essential for the maturation of the ribosomal 60S subunit. Functional studies in yeast and mammalian cells showed that altogether with Nop7 and Ytm1 it forms a stable subcomplex called PeBoW that is crucial for a correct rRNA processing. The exact function of the protein within the process remains unknown. The N-terminal region of the protein includes a well conserved region shown to be involved in PeBoW complex formation whereas the carboxy-terminal half was predicted to contain seven WD40 repeats. This first structural report on Erb1 from yeast describes the architecture of a seven-bladed β-propeller domain that revealed a characteristic extra motif formed by two α-helices and a β-strand that insert within the second WD repeat. We performed analysis of molecular surface and crystal packing, together with multiple sequence alignment and comparison of the structure with other β-propellers, in order to identify areas that are more likely to mediate protein-protein interactions. The abundance of many positively charged residues on the surface of the domain led us to investigate whether the propeller of Erb1 might be involved in RNA binding. Three independent assays confirmed that the protein interacted in vitro with polyuridilic acid (polyU), thus suggesting a possible role of the domain in rRNA rearrangement during ribosome biogenesis.

## Introduction

Erb1/Bop1 is a eukaryotic protein that was firstly described as an evolutionary conserved factor involved in large ribosomal subunit biogenesis in yeast and mammals respectively [1,2]. Its function is essential in the processing of rRNA precursors that give rise to the mature 5.8S and 25/28S particles [1–3]. Knock-down of Erb1 impairs ribosome assembly, leading to accumulation of immature rRNA species in yeast, whereas the overexpression of Bop1 negatively affects cell proliferation in mammals [1,3,4]. In addition, an N-terminally truncated mutant of Bop1 is able to induce a reversible growth arrest through p53 response, suggesting a possible role of the protein in ribosome biogenesis control [1,3]. Moreover, over-expression of *bop1* increases the number of multipolar spindles, implying a correlation with colorectal cancer [5].

In *Saccharomyces cerevisiae* Erb1 contains 807 residues and carries a well conserved N-terminal domain called BOP1NT which plays role in the recruitment of the protein to pre-ribosomes [6]. The C-terminal region of Erb1 was predicted to contain seven WD repeats that form a β-propeller domain of unclear function [1]. Additional work on the exact role of Erb1 in ribosome assembly showed that it formed part of a functional cluster of processing factors, called A3, that were responsible for the cleavage of ITS1 (Internal Transcribed Spacer 1) [7,8]. It has been also demonstrated that the full length protein binds to Domain I of 25S rRNA [9]. Erb1 directly interacts with Nop7 and Ytm1 proteins (Pes1 and Wdr12 in mammals, respectively) forming Nop7 sub-complex (called PeBoW in mammals) that co-purifies with pre60S particles but remains stable even after its dissociation from pre-ribosomes [7,10,11]. Nop7 complex has to be removed from the nascent ribosome by the AAA-ATPase Rea1 in order to promote normal ribosome maturation [12]. Since Ytm1 and Nop7 do not physically interact, Erb1 is considered to be the core of the complex and the ratio of Nop7/Erb1 and Erb1/Ytm1 heterodimers is important in controlling the assembly and function of Nop7 complex (as shown for PeBoW complex in mammals by Rohrmoser [4]). The involvement of the complex in ribosome biogenesis was reviewed by Henras [13].

While several studies regarding Erb1 function and interactions focus on the BOP1NT domain, the role of the propeller is still under investigation. It was shown that in yeast a truncated Erb1 lacking the C-terminal domain would not cause growth arrest but presented only a mild defect in rRNA processing [6]. Despite the fact that the β-propeller domain of Erb1 has been proposed as dispensable for ribosome assembly, it still presents a high degree of conservation in all eukaryotes. It is worth noting that the binding partner of Erb1, Ytm1 is also predicted to contain a large 7-bladed β-propeller region on its C-terminus [14]. Furthermore, there are described additional 20 proteins that contain β-propeller domains in their structures and form part of the ribosome assembly pathway in eukaryotes, thus indicating that it is a common fold required to establish a high-affinity protein-protein interaction network within this complex pathway [13].

In recent years, there has been an increasing interest in the architecture of pre-ribosomes in order to get a better understanding of the dynamics of the process. However, there is very limited amount of information regarding the pre-ribosomal particles from a structural point of view. The main challenge in the field is the lack of stability of the individual components of this enormous machinery as well as the difficulty when trying to obtain homogenous samples for structural studies [15]. Thanks to the recent advances in cryo-EM technique it has been possible to get an insight into the organization of the late-stage pre-ribosome, nevertheless the structure of the majority of the factors that participate in ribosome maturation still remains unknown [16,17].

Here we present the structure of the β-propeller domain of Erb1 at 1.6Å resolution that was obtained during crystallization trials of Erb1/Nop7 dimer. The structural information allows us to exactly define the boundaries of the domain and to describe its particular features, being the presence of a long insertion within the second WD repeat the most distinctive characteristic. We consider a possible role of this extra fold in protein-protein interactions based on its importance for crystal packing. At the same time, surface analysis helps us to predict other areas that are likely to be involved in recognition of proteins or nucleic acids therefore making the C-terminal domain of Erb1 a motif capable of binding to additional factors within pre60S network. At last, we demonstrate that, indeed, the β-propeller of Erb1 is able to bind non-specifically RNA in vitro through a saturable surface.

## Materials and Methods

### Cloning

Since the genes of *nop7* and *erb1* do not contain introns, they were cloned from the genomic DNA of *Saccharomyces cerevisiae*. A second PCR was performed in order to add overhangs suitable for ligase independent cloning (LIC) cloning and compatible with vectors of interest. *Nop7* was cloned into pNIC28-Bsa4 vector which contains a sequence for N-terminal 6xHis tag followed by TEV (Tobacco Etch Virus protease) cleavage site. E*rb1* was introduced into pET28-NKI/LIC 6His/3C vector obtained from Dr A. Perrakis group (NKI, Amsterdam), containing the N-terminal 6xHis tag followed by 3C protease (Human Rhinovirus protease) cleavage site. Both ligation reactions were performed according to the standard LIC protocol using T4 DNA polymerase from Fermentas. The DNA coding for yeast *Erb1*_*518-586*_ containing LIC suitable overhangs was purchased from Life Technologies and the gene of Erb1_432-801_ from *Chaetomium thermophilum var thermphilum* (ChErb1) was cloned from the cDNA library prepared as described in [18]. Both genes were cloned into pET28-NKI/LIC 6His/3C as described above.

### Protein expression and purification

The 6xHis-Nop7 was expressed in BL21 Codon Plus strain of *E*. *coli* grown in LB. The expression was induced with 0.5mM IPTG and the culture was incubated overnight at 20°C. *E*. *coli* BL21 Codon Plus strain was also used for the expression of 6xHis-Erb1. Protein production was performed overnight at 20°C using ZY medium and autoinduction strategy described by Studier [19]. After 16h of expression the cells were collected by centrifugation and the resulting pellets were frozen in liquid nitrogen and stored at -80°C. Both proteins were purified separately following a protocol that involved IMAC (Immobilized-Metal Affinity Chromatography) and size exclusion chromatography. The cell pellet was resuspended in 40ml of lysis buffer containing 50mM Hepes pH 7.5; 0.5M NaCl; 10% glycerol; 5mM β-mercaptoethanol and 10mM of imidazole. One pill of Roche EDTA-free protease inhibitor cocktail was added to the buffer. The cells were disrupted by sonication and the soluble fraction was separated from the debris by centrifugation at 4°C/20000 x g for 45 minutes. The supernatant was filtered and loaded onto a 5ml HisTrap column (GE Healthcare) previously equilibrated with 10 volumes of Wash Buffer (20mM Hepes pH 7.5; 0.5M NaCl; 2mM β-mercaptoethanol; 5% glycerol and 20mM imidazole). The wash and elution steps were performed using the AKTA purification system (GE Healthcare) by following absorbance at 280 nm. First, the column was washed with 10 column volumes of Wash Buffer and then the protein was eluted in 5ml fractions using a step gradient of 3%, 12%, 40% and 100% of elution Buffer (20mM Hepes pH 7.5; 0.5M NaCl; 2mM β-mercaptoethanol; 5% glycerol and 500mM imidazole). The fractions containing the protein of interest were concentrated and injected into a Superdex200 16/60 column which was equilibrated with 1.5 column volume of SE buffer (20mM Hepes pH 7.5; 0.25M NaCl; 2 mM β-mercaptoethanol, 5% glycerol). The 5ml fractions were collected and those containing non-aggregated protein were concentrated.

Expression and purification of ChErb1_432-801_ were performed as described for Erb1from *S*. *cerevisiae* except for the size exclusion chromatography step. The buffer used for ChErb1_432-801_ was 50mM Tris pH8; 100mM NaCl and 5mM MgCl_2_. The fractions containing the protein were concentrated up to 4mg/ml.

### Protein expression and purification of Erb1_518-586_

The 6xHis-NKI was expressed in C41 (Lucigen, USA) strain of *E*. *coli* grown in 5l of LB. The expression was induced with 1mM IPTG, when the dispersion at 600 nm reached a value of 0.6–0.8, and the culture was incubated overnight at 37°C. After 16h of expression the cells were collected by centrifugation and the resulting pellets were frozen in liquid nitrogen and stored at -80°C. The cell pellet was resuspended in 50ml of lysis buffer (20 mM Tris pH 8.0; 0.5 M NaCl; 1mM β-mercaptoethanol; 0.1% Triton X-100 and 5mM of imidazole), supplemented with one tablet of Sigma protease inhibitor cocktail (EDTA-free). The cells were disrupted by sonication (10 burst of 45 s at the maximum power of the sonicator, Model 102-C, Branson, USA), in ice, and the soluble fraction was separated from the debris by centrifugation at 4°C/20000g for 45 minutes. The supernatant was filtered and added to a 5 ml of His-select nickel affinity gel (Sigma). The resulting mixture was incubated for 30 minutes at 4°C; after that time it was added to a Biorad empty column and the supernatant was separated by gravity. The resin was washed with 20 ml of 20 mM Tris pH 8.0; 0.5 M NaCl; 1mM β-mercaptoethanol and 25 mM of imidazole for 10 minutes. The supernatant was removed from the column by gravity. The protein was eluted from the resin with 20 ml of 20 mM Tris pH 8.0; 0.5 M NaCl; 1mM β-mercaptoethanol and 500 mM of imidazole. The presence of the protein in the eluate was confirmed by 18% SDS-PAGE gels. The protein was concentrated in Amicon centrifugal devices (Amicon, MW cutoff 3000 Da) and loaded into a gel filtration column Superdex 75 16/600 (GE Healthcare) coupled to a FPLC purification system (GE Healthcare), by following the absorbance at 280 nm. The column was equilibrated in buffer 50 mM Tris (pH 7.5) with 150 mM NaCl. For ^15^N-labelled samples, the M9 minimal medium was used to express the protein. Purification protocols were carried out as in LB medium.

### Complex formation and co-crystallization

After the size exclusion chromatography step, concentrated samples of Nop7 and Erb1 were mixed in equimolar amounts and injected into Superdex200 16/60 column equilibrated with SE Buffer. The 5ml fractions corresponding to the heterodimer were mixed, concentrated and used for crystallization. Initial crystallization trials were performed at 21°C, the concentration of the Nop7/Erb1 complex was 80mg/ml and drops containing 0.3μl of protein sample and 0.3μl of reservoir were set up. Crystals diffracting up to 2.9Å were obtained in 0.1M Hepes pH 7.5; 10% Polyethylene glycol8000 and 8% ethylene glycol. In order to improve crystal size and resolution we performed an optimization screen based on the Hampton Additives kit, the protein concentration used was 60mg/ml and the drop size of 0.5μl for protein sample, 0.5μl for reservoir and 0.1μl of additive was added.

### Data collection and processing

Crystals obtained in the original screening and from the Hampton Additives screening were flash-cooled in liquid nitrogen and diffracted at Diamond Light Source (Harwell, UK) I24 and I03 beamlines. The maximum diffraction up to 1.6Å was obtained with crystals grown with addition of 30% of ethanol The crystals contained one molecule per asymmetric unit and the space group was P 21 21 21 with the following unit cell parameters: a = 52.026Å, b = 62.432Å, c = 158.22Å; α = β = γ = 90.00°. The data were processed using XDS, merged and scaled in CCP4 [20,21]. Molecular replacement was done in parallel using MR module of Phenix and Balbes on-line MR suite [22,23]. The search model used in phenix.mr was a poly-Ala β-propeller based on the input model chosen by Balbes database search (PDB: 2H13), which corresponds to an unrelated WD40 protein, WDR5, engaged in histone binding. Obtained model and initial phases were then used for additional model building cycle by AutoBuild module from Phenix. The final structure was then refined combining phenix.refine suite and manual refining in Coot until the final r factors were R = 16.0% and R free = 17.4%. Data collection and refinement statistics are shown in Table 1. The model and structure factors were deposited in Protein Data Bank with PDB ID: 4U7A.

**Table 1:** Data collection and refinement statistics.

**Wavelength (Å)**	0.9763
**Resolution range (Å)**	58.07–1.6 (1.657–1.6)^a^
**Space group**	P 21 21 21
**Unit cell**	
**a, b, c (Å)**	52.02, 62.43, 158.22
**α, β, γ (°)**	90, 90, 90
**Total reflections**	446057 (43775)
**Unique reflections**	67582 (6545)
**Multiplicity**	6.6 (6.7)
**Completeness (%)**	98.07 (96.78)
**Mean I/sigma(I)**	19.48 (2.16)^b^
**Wilson B-factor**	22.85
**R-merge**	0.04807 (0.8424)^c^
**R-meas**	0.0523
**CC1/2**	0.999 (0.834)
CC*	1 (0.954)
**R-work**	0.16 (0.27)^d^
**R-free**	0.17 (0.29)^e^
**Number of non-hydrogen atoms**	3302
**macromolecules**	2928
**ligands** ^f^	17
**water**	357
**Protein residues**	356
**RMSD** ^g^ **(bonds)**	0.006
**RMSD** ^g^ **(angles)**	1.07
**Ramachandran favored (%)**	97
**Ramachandran outliers (%)**	0
**Average B-factor (Å)**	31.10
** Macromolecules (Å)**	30.00
** Ligandsf (Å)**	44.60
** Solvent (Å)**	39.10

^a^ Statistics for the highest-resolution shell are shown in parentheses.

^b^ Mean [*I*/*σ*(*I*)] is the average of the relation between the intensity of the diffraction and the background.

^c^ R_meas_ = {Σ_*hkl*_ [N/(N-1)]^1/2^ Σ_i_ |*I*_*i*_(*hkl*)—<*I*(*hkl*)>|} / Σ_*hkl*_ Σ_i_ *I*_*i*_(*hkl*), where *I*_*i*_(*hkl*) are the observed intensities, <*I*(*hkl*)> are the average intensities and N is the multiplicity of reflection *hkl*.

^d^ R-work = Σ_*hkl*_ {[*F*_*obs*_(*hkl*)]—[*F*_*calc*_(*hkl*)]} / Σ_*hkl*_ [*F*_*obs*_(*hkl*)], where *F*_*obs*_(*hkl*) and *F*_*calc*_(*hkl*) are the structure factors observed and calculated, respectively.

^e^ R-free corresponds to R_factor_ calculated using 2% of the total reflections selected randomly and excluded during refinement.

^f^ Ligands: glycerol, ethylene glycol, ethanol.

^g^ RMSD is the root mean square deviation.

### Circular dichroism

Spectra of Erb1_518-586_ were collected on a Jasco J810 (Japan) spectropolarimeter connected to a Peltier unit. The instrument was periodically calibrated with (+)-10-camphorsulfonic acid. Spectra were acquired at 25°C in phosphate buffer at pH 7.0 (10mM). For each experiment, corresponding blank solutions were subtracted. The response time was 2 s, and experiments were averaged over 6 scans, with a scan speed of 50 nm/min. The step resolution was 0.2 nm, and the band-width was 1 nm. Molar ellipticity was obtained as described [24]. We explored a wide range of protein concentrations (10–80 μM) to ascertain whether shape or intensity of the far-UV CD (circular dichroism) spectra were protein-concentration dependent; in that range, we did not observe variation in any spectral parameter. The cell path-length was 0.1 cm. Every experiment was repeated three times with new samples.

### Fluorescence

Spectra were collected on a Cary Eclipse spectrofluorometer (Agilent) interfaced with a Peltier system at 25°C. A 1-cm-path-length quartz cell (Hellma) was used. The proper blank solutions were subtracted in all cases. Samples were prepared by taking the corresponding amount of a concentrated stock solution of Erb1_518-586_ protein to yield a final protein concentration of 1μM. Spectra of Erb1_518-586_ in aqueous solution (pH 7.0, 10mM Hepes and 0.150M NaCl) were acquired by excitation at 280 and 295nm; the emission spectra were collected between 300 and 400 nm. The excitation and emission slits were set to 5 nm, and the response was set to 1 nm.

### NMR spectroscopy

The 1D-^1^H-NMR and 2D- (^1^H, ^15^N) HSQC experiments of *Erb1*_518-586_ were acquired at 25°C on a Bruker Avance DRX-500 spectrometer (Bruker GmbH, Germany), equipped with a triple resonance probe and z-pulse field gradients. Processing of spectra was carried out with TOPSPIN software. Spectra were calibrated with external TSP for the ^1^H dimension, and for the ^15^N dimension as described [25]. All spectra were acquired in phosphate buffer (pH 7, 50 mM).

#### 1D-1H-NMR experiments

Protein concentration was 80 μM. Water was suppressed with the WATERGATE sequence [26]. A number of 512 scans were acquired with a spectral width of 12 ppm, with 16 K data points in the time domain. The data matrix was zero filled to 32 K during processing.

#### 2D 15N-HSQC experiments

Spectra of ^15^N-labeled *Erb1*_*580-586*_ were acquired in the phase-sensitive mode; frequency discrimination in the indirect dimension was achieved by using the echo/antiecho-TPPI method. The 2D ^15^N-HSQC experiment [27] was acquired with 4K data points in the ^1^H dimension and 200 scans in the ^15^N axis. The spectral widths were 15 and 35 ppm in the ^1^H and ^15^N dimensions, respectively. Carrier frequency of ^1^H was set at 4.8 ppm and that of ^15^N was 120 ppm. Water was suppressed with the WATERGATE sequence [26]. The concentration of *Erb1*_*580-586*_ was 200 μM.

### In vitro RNA binding assays

#### Poly(U)-agarose beads binding

Approximately 20μl of the polyuridylic acid-agarose (polyU) were used by assay. The beads were equilibrated 5 times with 500μl of reaction buffer each (50mM Tris pH 8; 100mM NaCl; 5mM MgCl_2_; 3mM DTT; 0.1% Triton-X100 and 0.1 mg/ml BSA) then 500μl of the sample containing 200μg of protein diluted in the reaction buffer were loaded on the beads and incubated at room temperature for 30 minutes. Unbound protein was removed by washing the beads five times with wash buffer (50mM Tris pH 8; 100mM NaCl; 5mM MgCl_2_; 3mM DTT; 0.1% Triton-X100). The bound fraction was eluted by addition of 30μl of 6x loading buffer and boiling for 5 minutes at 95°C. The samples were then analyzed on 10% polyacrylamide gel. The saturation was studied by incubating the protein sample with 0.1mg/ml or 1mg/ml of free polyuridylic acid for 30 minutes before it was loaded onto the beads. 1mg/ml of heparin was added to the protein sample prior to the poly(U) agarose binding in order to estimate the strength of the interaction.

#### Determination of the ChErb1432-801-polyU binding constant

Spectra of isolated ChErb1_432-801_ and of the complexes of ChErb1_432-801_/polyU, at different polyU concentrations, were acquired by excitation at 280 or 295 nm; the emission was collected between 300 and 400 nm. The excitation and emission slits were set to 5 nm, and the response was 1 nm.

Binding experiments of the polyU to ChErb1_432-801_ were carried out as described [28]. Briefly, increasing amounts of polyU, in the range 0.5–5 μM, were added to a solution containing 1.5 μM of ChErb1, in 20 mM Hepes (pH 7.5), 150 mM NaCl, 5% glycerol and 2 mM ß-mercaptoethanol; the fluorescence was measured at 25°C, after preparing the samples. The apparent dissociation constants of the ChErb1-polyU complex, KD, was calculated by fitting the fluorescence change in intensity [29], versus the concentration of added polyU to:

$$F_{meas}=F+\Delta F_{\max}\left(\left(\left[polyU\right]+\left[ChErb1\right]+K_{D}\right)-\sqrt{{\left(\left[polyU\right]+\left[ChErb1\right]+K_{D}\right)}^{2}-4{\left[ChErb1\right]}^{}\left[polyU\right]}\right)$$ (1)

where [polyU] is the concentration of ribonucleic acid; [ChErb1] is the concentration of the protein; Fmeas is the measured fluorescence parameter at each concentration of added polyU; ΔFmax is the change in that parameter, when all ChErb1_432-801_ is forming the complex; and F is the fluorescence parameter of the mixture of the complex Fitting to Eq (1) was carried out with Kaleidagraph (Abelbeck software).

#### Biolayer interferometry

Dissociation constant (KD) was determined more accurately by BioLayer Interferometry using BLItz system (ForteBio). A sample containing 50μg/ml of 15 nucleotide-long 5’-biotinylated polyU from Sigma-Aldrich was immobilized on Streptavidin biosensors (Forte Bio) previously hydrated with sample buffer (50mM Hepes pH 7.5; 150mM NaCl; 5% glycerol and 2mM β-mercaptoethanol). Increasing amounts of ChErb1_432-801_ (0μM; 2μM; 5μM; 7.5 μM; 15 μM and 23μM) were used in association and dissociation steps. Curve fitting of triplicates and K_D_ calculation were carried out with BLItz Pro 1.2 software.

## Results

### Crystal unit cell suggests crystallization of a fragment produced by proteolytic cleavage

Initial analysis of the diffraction data showed that the asymmetric unit volume was 125433.9Å^3^, not large enough to accommodate Erb1/Nop7 complex nor Erb1 alone. For Nop7 alone the Matthews coefficient and solvent content were 1.78Å^3^/Da and 31% respectively, lower than expected [30]. In addition, the Xtriage module from Phenix estimated 470 residues as the most probable in the asymmetric unit which led us to investigate whether the full length Nop7 or a fragment of any of the two components had been crystallized.

**Fig 1:**
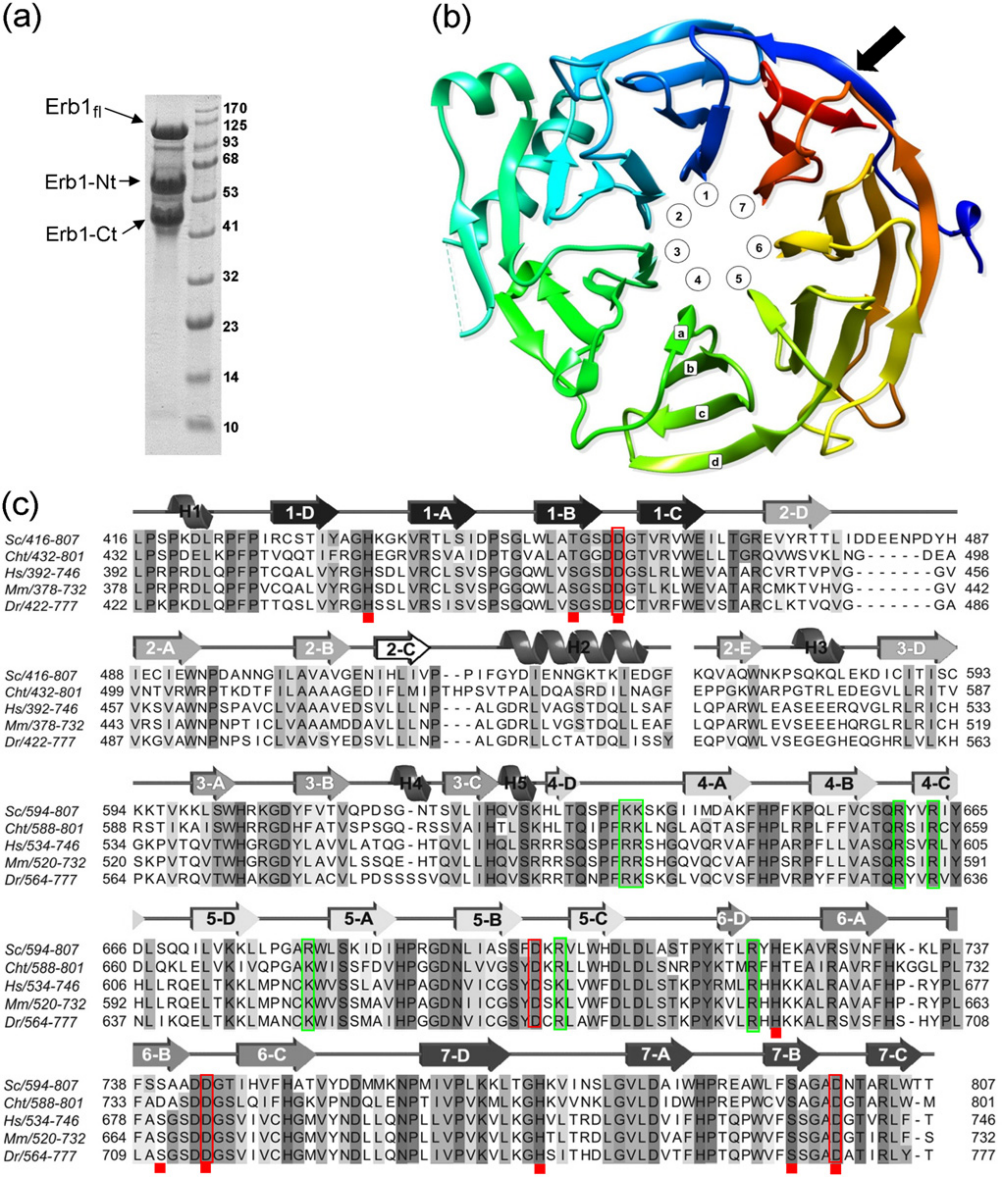
Erb1 degradation and β-propeller general features. (a) Coomassie—stained SDS-PAGE showing a severe degradation pattern of full-length Erb1 when incubated at 4°C overnight. The N-terminal and C-terminal degradation products are marked. MW in kDa is shown next to the ladder. (b) Ribbon representation of the β-propeller domain of Erb1 seen from the top (by convention the top face is described as the one that contains loops B-C and D-A). The blades numbering is counterclockwise and the β-strands nomenclature follows as shown for blade 4. Black arrow indicates the Velcro-like closure of the domain. (c) Sequence multi alignment of the carboxy-terminal domain of Erb1/Bop1 from *Saccharomyces cerevisiae* (Sc), *Chaetomium thermophilum* (Cht), *Homo sapiens* (Hs), *Mus musculus* (Mm) and *Danio rerio (Dr)*. For clarity only the residues present in the final pdb model are shown. Conserved amino acids are marked with shadows. Secondary structure assignment is shown on the top of the alignment. Numbers of β-strands correspond to the WD repeats of the protein. Red rectangles mark conserved Asp in B-C loops and red squares indicate residues forming His-Thr/Ser-Asp triads. Green rectangles show basic residues that form a putative RNA-binding site.

In order to confirm if the crystals from initial screenings contained Nop7 or a fragment of Erb1, 15 crystals were analyzed by SDS-PAGE and, after staining with Coomassie Blue, only a single faint band of approximately 45 kDa could be observed on the gel. When the stability of Nop7, Erb1 and the Nop7/Erb1 complex was assayed, it was seen that after 24h of incubation at 4°C Erb1 started to show a clear pattern of degradation that was visible even upon binding to Nop7 and led to the apparition of the 45kDa band as seen for the crystals (Fig 1a). The mass-spec analysis confirmed that the lower MW band corresponded to the C-terminal region of Erb1 and contained the whole β-propeller domain of the protein.

### The carboxy-terminal domain of Erb1 is a 7 bladed β-propeller

**Fig 2:**
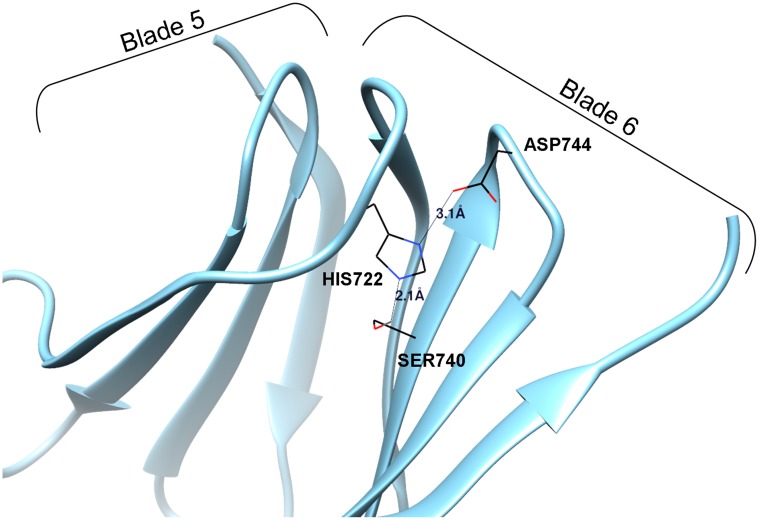
A canonical Asp/His/Ser triad necessary for correct blade organization. Conserved residues participating in the crucial hydrogen bond formation are shown and labelled. The distances between the atoms directly involved in H-H bonding are also represented.

The solved structure of the C-terminal domain of Erb1 has confirmed that it folds into a seven-bladed β-propeller as previously predicted [1]. The blade organization and the nomenclature are shown on the Fig 1b and 1c. Structure analysis led us to slightly extend exact boundaries of the domain (residues 427–807) towards the N-term, when compared to the sequence-based prediction (residues 435–807) because the strand D (outermost) of blade 7 (most C-terminal) is actually formed by residues 427–433 which participate in the “velcro-like” closure of the domain (Fig 1b). Curiously, none of the WD repeats contains the eponymous WD motif but they rather present HD/YD dipeptides at the end of the strand C. Similarly to other WD40 domains, no clear pattern can be observed between the sequences of the repeats, although the hydrophobic core of the domain is well conserved and forms intra-molecular interactions necessary for proper folding. One of the most conserved features common for the β-propeller folds is the presence of a non-variable Asp in the loops that connect strands B and C of each blade. This residue is involved in stabilization of the domain as it forms a triad with a conserved His from the GH motif and a Ser/Thr residue placed in strand B. In Erb1, five of B-C loops contain an Asp residue but only four of them are truly conserved (red boxes Fig 1c) and the triad appears only in blades 1, 6 and 7 (Fig 2 and red squares in the alignment from Fig 1c). In loop 2B-2C there is a glutamic acid (Glu508) and in loop 4B-4C a glutamine (Gln659), both are conserved and establish a network of interactions that stabilizes the folding but is not similar to the canonical Asp-His-Ser/Thr triad. In the third blade, non-conserved Asp615 seems to be important for inter-blade interactions as it establishes hydrogen bonds with neighboring residues (Lys595 and Ile641), but it is rather to be substituted by a Gln in higher eukaryotes (Gln556 in human Bop1). It has been proposed that the conservation of the Asp residue could be correlated with the interacting His which means that if a WD repeat lacks the Asp it would also loose the His during evolution [31]. This seems to be true for Erb1 as the His from D-A loop is only conserved in the three repeats that form the triad with Asp. At last, Asp701 from B-C loop of blade 5 is fully conserved but the His is not present, thus the triad cannot be formed. Instead, this residue altogether with conserved Arg703 makes electrostatic and hydrogen-bonds that stabilize the fifth blade.

The overall architecture of Erb1Ct superposes well with other 7-bladed propellers and is structurally highly similar to the β-propeller domain of the transducin-like enhancer protein1 (PDB: 2CE9) with the RMSD (Root-Mean-Square Deviation) score of 2.2Å as calculated by Dali Server [32]. The main difference between these characteristic propeller folds lies in the presence of loops that connect β-strands and contribute to the functional specialization of the domain by creating surfaces that will enable specific protein-protein interactions. Regarding Erb1, some of these loops are quite long and might be very flexible as reflected by high temperature factors corresponding to the residues 478–488 that connect strands 2D and 2A, nevertheless when compared to Erb1/Bop1 in higher eukaryotes, a clear tendency to loop shortening may be observed (Fig 1c). Interestingly, we detected a big variation regarding the conservation of the loops that connect the blades. In general, the loops between strands A-B (bottom side of the propeller) are more variable in sequence, whereas those on the top face, connecting strands B and C (which contain Asp residues highlighted in Fig 1c), present a higher degree of conservation thus indicating an important region for the β-propeller stability or a conserved protein-protein interaction surface.

**Fig 3:**
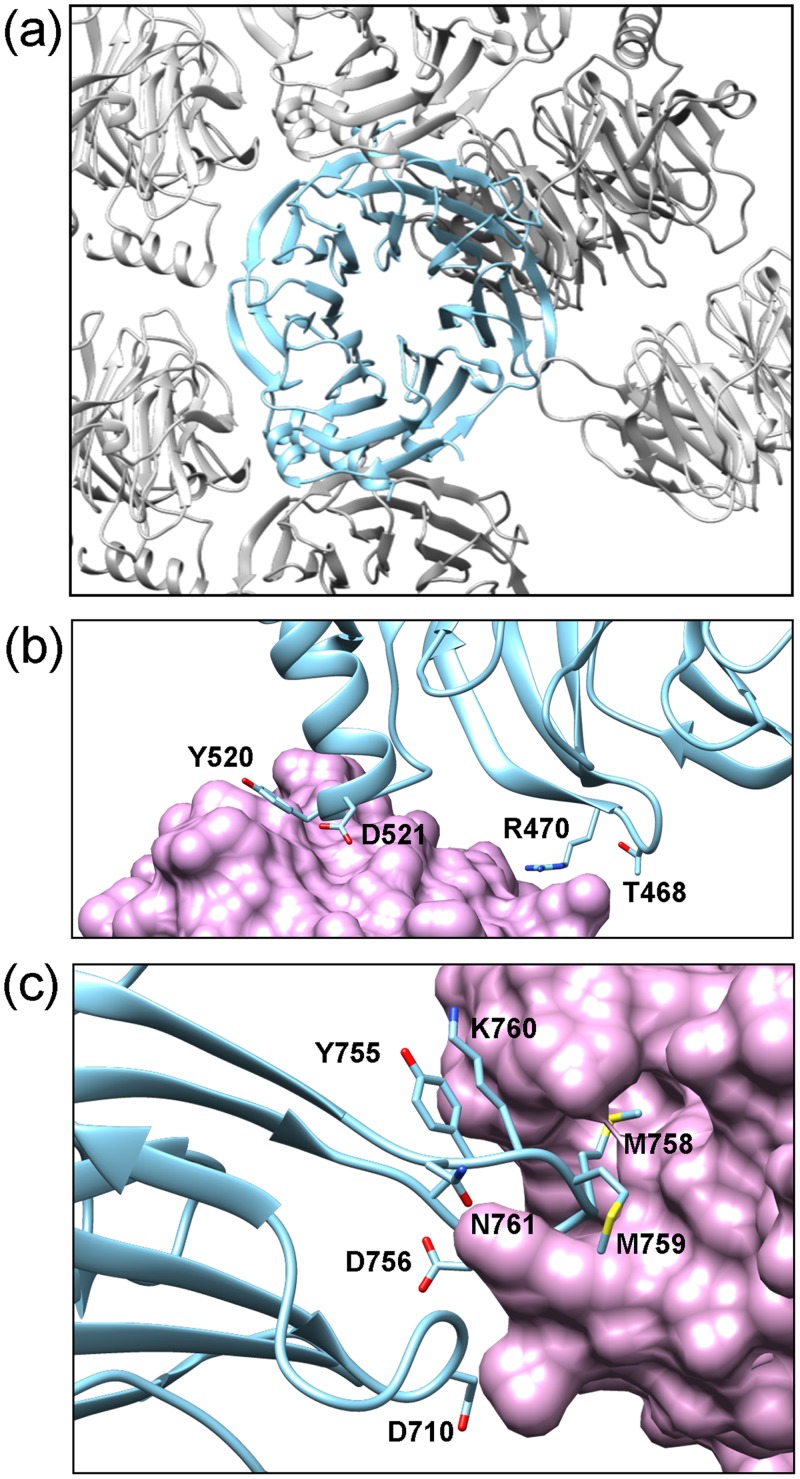
Analysis of crystal packing. (a) Overview of crystal contacts of Erb1 monomer (blue) with symmetry related molecules (grey) shows that the top and bottom areas of the propeller are not involved in crystallographic interactions. (b) Helix H2 interacts with symmetrically related molecule (shown in pink). (c) 6C-7D loop penetrates deeply into a conserved cavity of another monomer (in pink). The residues directly involved in crystal packing are labelled in (b) and (c).

From a crystallographic point of view, unlike in several crystal structures of β-propellers [33–35], the extensive top or bottom areas of the domain do not form many contacts with symmetry-related molecules but the interactions are rather maintained laterally through the outermost loops and strands (Fig 3a). Manual inspection of the unit cell and analysis by Pisa Server [36] showed that a single monomer contacted six other molecules making three small interfaces involved in crystal packing. Two of them arose in the bottom part of the propeller where the alpha-helix H2 orientates in proximity of blades 1 and 7 from one symmetry related monomer and the loops 5B-5C and 6D-6A from another propeller (Fig 3b). The third area corresponds to the long 6C-7D loop which forms an important extension and introduces Asp757, Met758 and Met759 into a cavity formed between Phe635, Tyr665 and Gln670 from blades 3 and 4 (Fig 3c). Both the loop and the cavity are well conserved within Erb1 family and may constitute additional elements involved in protein binding. The central axis of the propeller is filled with solvent (water) molecules.

### The β-propeller domain of Erb1 contains a long insertion within blade 2

**Fig 4:**
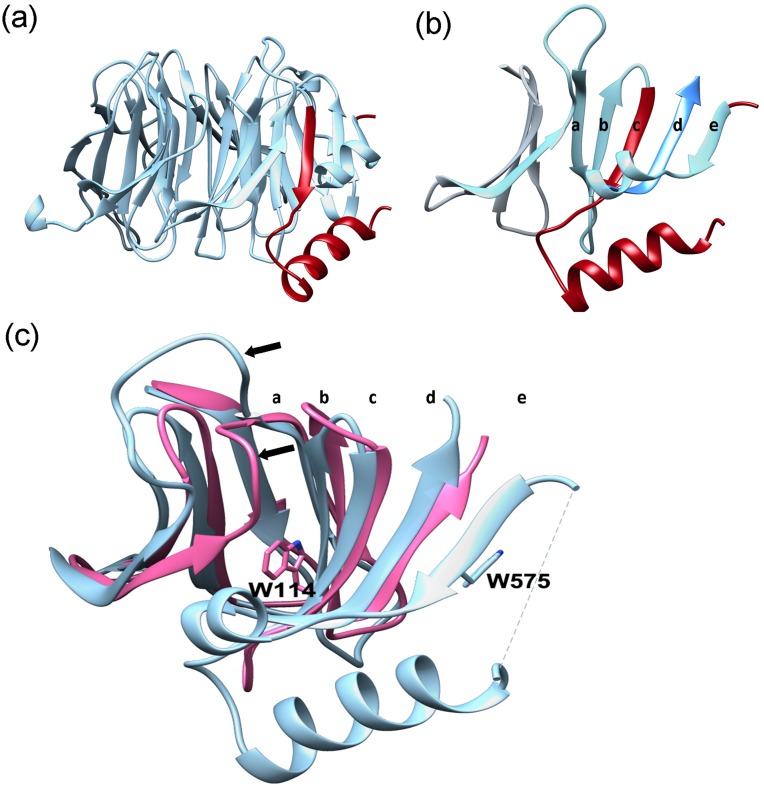
Insertion within WD repeat 2. (a) The insertion (red) forms an important protrusion on the bottom of the domain. (b) Position of the insertion (red) in the context of the second blade only. Residues corresponding to WD repeat 2 are represented in light blue and the strand D of WD repeat 3 is shown in dark blue. (c) Comparison of blades 1 and 2 of WDR5 from *H*. *sapiens* (PDB:4CY2; pink) and Erb1 (blue). Side chains of conserved tryptophan corresponding to the strand C (in canonical WD repeats) are shown for both proteins. Black arrows indicate the position of 2D-2A loops. The letters in (b) and (c) indicate the position of each strand.

Undoubtedly, the most distinctive feature of the core of the β-propeller in this study is the blade 2 which due to an insertion contains five, and not four, β-strands and shows a protrusion attributed to two α-helices (H2 and H3 on Fig 1c). Electron density map allowed us to trace and build model for residues 515–534 and 571–594, being the rest of the insertion unmodeled. This missing part seems to be Fungi specific as it becomes much shorter in higher eukaryotes (Fig 1c). Helix H2 (residues Tyr520-Asp532) appears between strands 2C and 2E and is attached to the base of the propeller (Fig 4a). In general, the sequence of the helix is poorly conserved, but it contains two invariable non-polar residues: Ile522 that makes hydrophobic contacts with the backbone of 2A-2B loop and Ile530 which interacts with the C-terminal fragment of strand 3D. The β-sheet corresponding to this blade is formed by strands 2A, 2B, 2C, 3D and 2E clearly indicating an alteration of a standard WD40 pattern (Fig 4b). Whereas strand 3D unambiguously indicates the beginning of WD3, the sequence of WD repeat 2 does not show any significant conservation but still contains strategic residues that allow formation of hydrophobic and electrostatic interactions with neighboring blades. Initial sequence-based analysis suggested that between WD repeats 2 and 3 there was an approximately 80-residue long segment which did not contain any WD pattern. Surprisingly, when we aligned the sequence of Ct domain of Erb1 with other non-Erb1/Bop1 β-propeller-containing proteins we could clearly see that Trp575 from strand 2E corresponded to Trp residue from WD dipeptide that typically appears in strand C (as in human WDR5 protein, PDB: 2H14) (Fig 4c) [37]. This fully conserved residue establishes important hydrophobic interactions with Ile592 from strand 2D and His629 located in 3D that are likely to be required for a proper conformation and attachment of the insertion to the side of the blade 2. We conclude that from an evolutionary point of view strand E corresponds to strand C from a canonical blade although displaced, in the second blade of Erb1, by a segment containing 2C-loop-H2. This insertion produced an important reorganization of the whole blade, altering the position and function of Trp-Asp dipeptide (Trp-Asn in this case). As a result, the second blade lacks the important Trp residue at the end of strand 2C that would guarantee correct approach between blades 1 and 2. We observe that in this case there is a different interaction network, conserved in Erb1/Bop1 family but not in other WD repeat-containing proteins, that involves strand 2D from blade 1 and a short α-helix, H3, from blade 2 (Gln580-Lys585). This helix inserts between strands 2E and 3D and possesses two non-conserved lysine residues (Lys581; Lys585) that interact with loop 2D-2A through hydrogen bonds. In consequence of this arrangement, α-helix H3 forms a lid that orientates close to a very hydrophobic area in blade 2 created by a segment of well conserved polar residues from strand 2B. It is important to keep in mind that loop 2D-2A is quite flexible and its vertical orientation makes the whole interface between blades 1 and 2 more opened when compared with the gaps between other blades which are completely covered by D-A loops (Fig 4c).

### The isolated insertion, Tyr518-Asp586 (Erb1_518-586_), is disordered in solution

**Fig 5:**
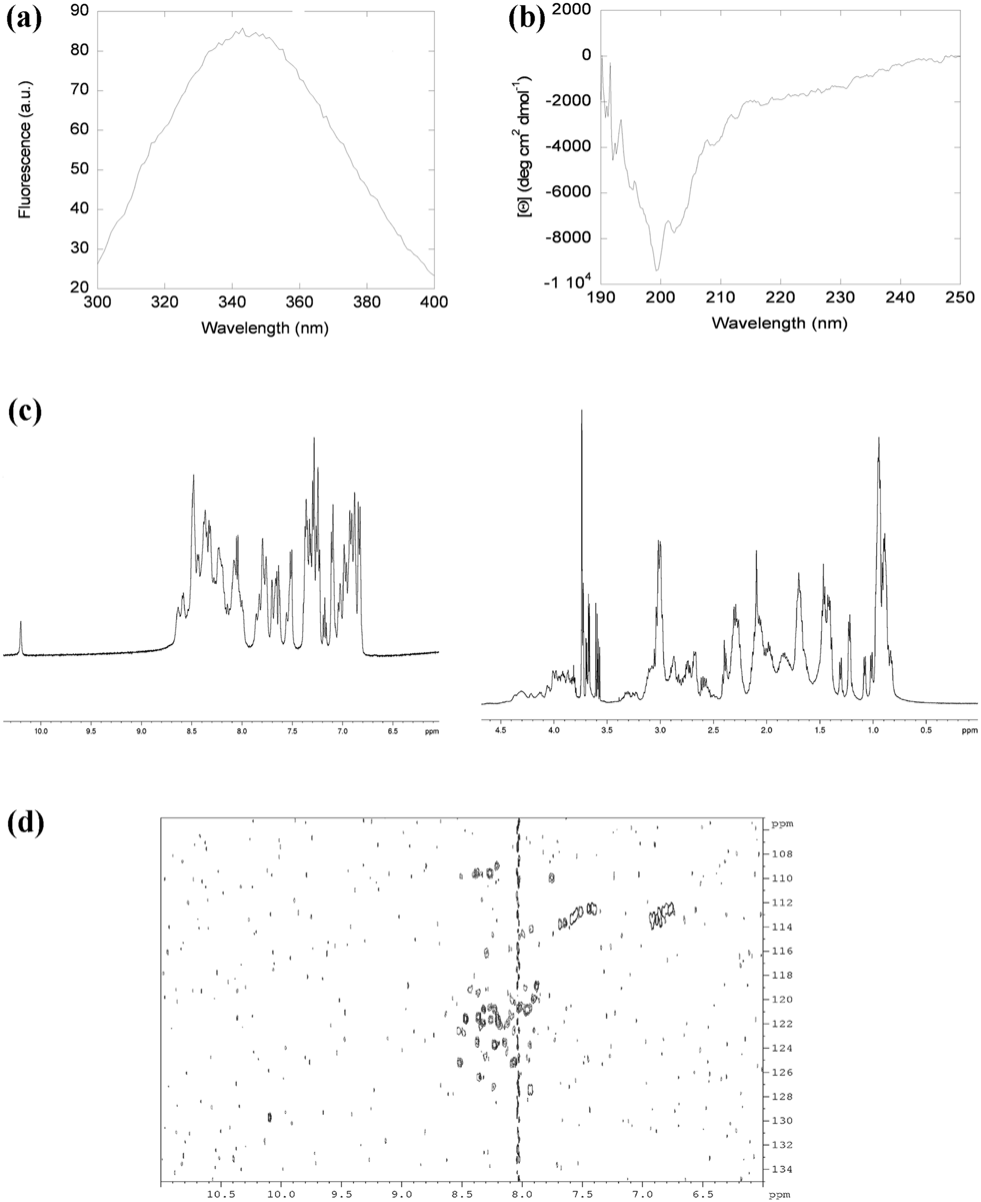
Erb1 insertion is disordered in solution. (a) Fluorescence spectrum of Erb1_518-586_ obtained by excitation at 280 nm. (b) Far-UV CD spectrum of Erb1_518-586_. (c) Amide (left) and alkyl (right) regions of the 1D-^1^H- NMR spectrum of Erb1_518-586_. (d)^15^N-^1^H HSQC spectrum of Erb1_518-586_.

We studied the conformational propensities of the isolated insertion, the fragment comprising residues Tyr518-Asp586 of Erb1, in solution. Fluorescence spectrum showed the maximum at 345 nm, suggesting that the sole Trp, Trp575, was highly solvent-exposed (Fig 5a). Furthermore, its far-UV CD spectrum had an intense minimum at 200 nm, and a shoulder at 222 nm (Fig 5b), which suggests the presence of helical or turn-like conformations. Those structured conformations, however, were not stable enough, since thermal denaturations followed by far-UV CD did not show any sigmoidal behavior (data not shown). The 1D-^1^H–NMR spectrum had all the amide protons clustered between 8.0 and 8.5 ppm. Moreover, the indole proton appeared at 10.19 ppm, close to the expected value of a random-coil indole moiety [38], and thus further supporting the fluorescence results. In the 1D-^1^H-NMR spectrum, there was no down-field shifted H_α_ protons (suggesting the absence of β-strands); however, there were a few up-field shifted methyl protons, which are probably close, in the primary structure, to some of the aromatic groups (Fig 5c). Finally, the 2D-^15^N-HSQC experiments (Fig 5d) had a very narrow dispersion in the amide proton region of 0.4 ppm that is centered around the random-coil chemical shift values for NHs (between 8.2 and 8.4 ppm), confirming the 1D-NMR results. In addition, there was a lower number of cross-peaks than we should expect (79); the absence of the peaks is either probably due to chemical exchange broadening or rapid amide exchange, as expected in natively unfolded proteins [39].

### Evolutionary conserved clusters on the surface of the propeller form putative ligand binding pockets

The best known and most obvious function of β-propellers is their capacity to establish multiple interactions with other proteins. The intrinsic rigidity and the shape of the domain create three well defined zones where the binding partner can attach: the top, the bottom and the circumference of the propeller. We searched for conserved residues on the surface of Erb1Ct which could indicate a region important to preserve protein interaction interface. There is a very clear division between a poorly conserved area, that includes blades 1, 2 and the upper part of blade 3, and a less variable surface of blades 4, 5, 6 and 7. In the bottom part, between blades 3 and 4 we identified a well conserved pocket which is a good candidate as a possible place of association with a binding partner.

**Fig 6:**
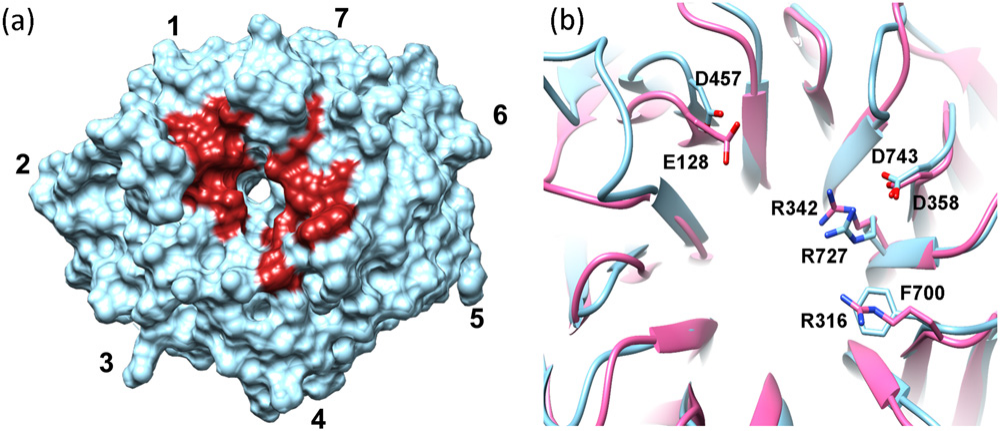
Top face of the β-propeller contains “hot spot” residues. (a) Top face of the propeller showing the position (in red) of the residues most likely to participate in macromolecular interactions as predicted by WDSP server (http://wu.scbb.pkusz.edu.cn/wdsp/). (b) Superposition of Lis1 (pink, PDB: 1VYH) with Erb1 β-propeller. The side chains of the conserved amino acids are shown and labeled.

In addition, it has been proposed that the central channel of WD domains could work as a scaffold that adapts for recognition of different ligands through side-chains of three residues from each blade: the one right before the strand A (A-1), the one just after strand B (B+1) and the second residue in the strand A (A2), thus making this portion of the propeller an universal but variable binding motif. When we inspected these positions in Erb1Ct a strong conservation, especially in blades 1, 5, 6 and 7, was observed. Our findings were confirmed by WDSP web server which predicted hot-spot residues on the surface that were likely to be responsible for high-affinity interactions with other proteins [40] (Fig 6a). Moreover, those conserved positions seem to be related to Erb1/Bop1 function because they vary when compared to seven-bladed propellers from other families. Nevertheless, three of these superficial conserved amino acids, Asp457, Arg727 and Asp743 are also invariable in another family of WD repeat-containing proteins called Lis1 where they were shown to be involved in recognition of other macromolecules [41] (Fig 6b).

### The carboxy-terminal domain of Erb1 binds RNA in vitro

**Fig 7:**
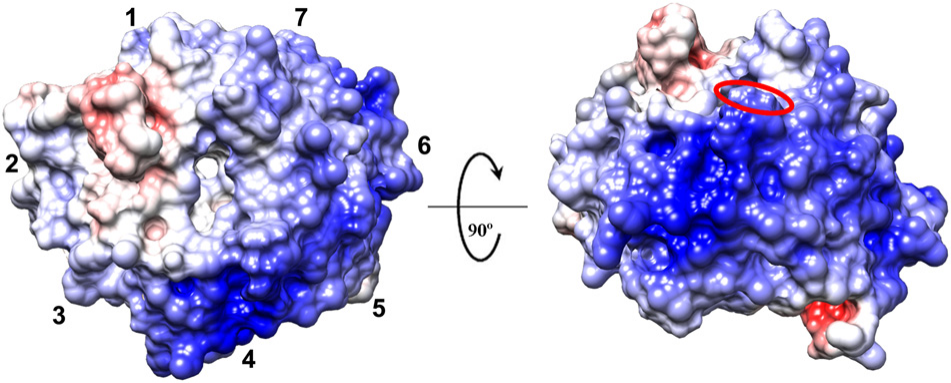
Surface of Erb1 β-propeller is positively charged. Surface representation of the electrostatic potential of the domain (from red (-10) to blue (+10)k_b_ T e_c_^-1^). The top face is shown on the left and the most positively charged area formed by blades 4 and 5 is visible on the right panel. The red oval indicates the position of Trp682.

Erb1 is known to bind rRNA as shown by UV-crosslinking experiments. It is possible that the β-propeller is involved in such a binding due to its highly positive charge (Fig 7). We used the PatchFinder Plus algorithm [42] to identify the biggest positive patch on the surface of the domain. Indeed, as seen for the electrostatic surface analysis, the tool found a big region of basic residues on the surface that included five blades and the entrance to the central channel on the top face of the propeller. Curiously, one of the few known structures of a WD domain bound to a nucleic acid is the one of DDB1-DDB2 complex with a DNA chain binding the protein through a cavity formed by the arginine and lysine residues oriented around the central tunnel of the propeller [43]. In Erb1 this area also contains well conserved basic amino acids: Arg441, Lys598 and Arg727.

**Fig 8:**
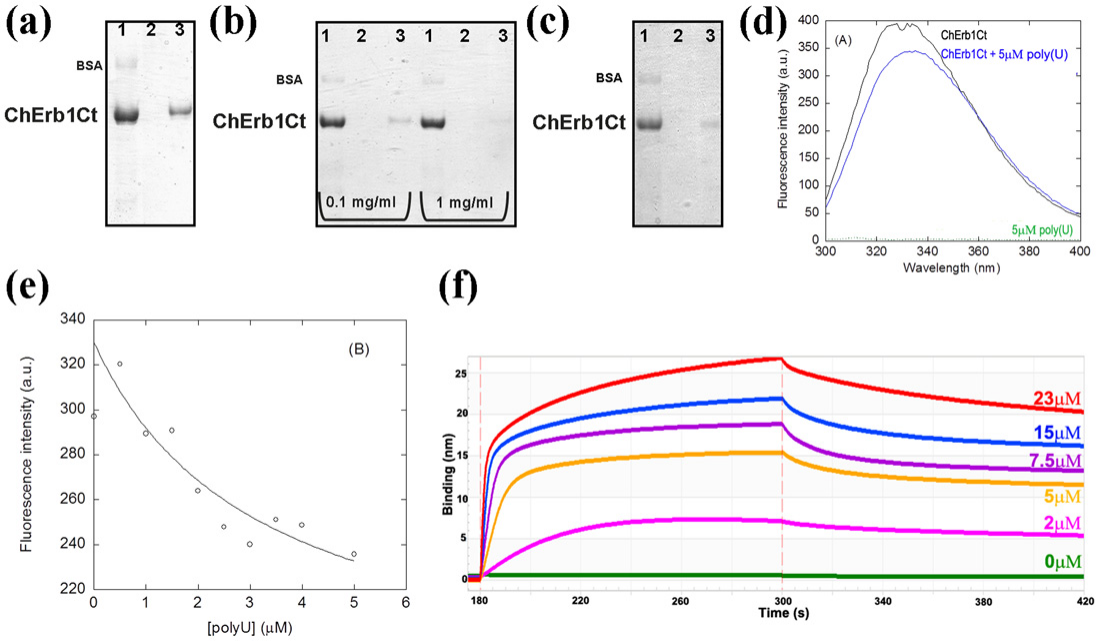
ChErb1Ct (residues 432–801) binds RNA *in vitro*. (a) Coomassie-stained SDS-PAGE showing the binding of Erb1 β-propeller from *Ch*. *thermophilum* to polyU agarose beads. (b) The saturation of the binding is visible upon addition of 0.1 mg/ml or 1 mg/ml of free polyuridilic acid. (c) The binding is still detectable upon addition of 1 mg/ml of heparin to the binding buffer. (a) (b) and (c) 1: Input, 2: Wash, 3: Elution; (d) Fluorescence spectra of ChErb1_432-801_ alone (in black) and with 5μM poly(U) (blue) obtained by excitation at 280 nm. The spectra were acquired at 25°C with 1.5 μM of ChErb1_432-801_. The green line at the bottom of the spectra is the emission spectra of polyU at a concentration of 5 μM. (e) Titration curve obtained from the emission fluorescence intensity at 315 nm. The line is the fitting to Eq (1). (f) Association and dissociation curves of 15nt-poly(U) with different concentrations of ChErb1_432-801_ measured by biolayer interferometry.

Initially, we checked the affinity of the β-propeller of Erb1 for RNA in vitro using poly(U) agarose beads. Our first attempts to perform in vitro binding assays failed because the C-terminal domain of Erb1 (residues 416–807) from yeast expressed poorly in *E*. *coli* and rapidly degraded during purification. We decided to try whether the same domain from a thermophile would be stable enough to carry out the experiments. Finally, a truncated Erb1 from *Chaetomium thermophilum*, comprising residues 432–801 (ChErb1_432-801_), was used for the assays due to its enhanced stability and higher expression levels in *E*. *coli*.Since the sequence of the domain is well conserved between *S*. *cerevisiae* and *C*. *thermophilum*, including the basic residues from the putative RNA-binding area (shown with green boxes in Fig 1c), we consider ChErb1_432-801_ to be suitable for validation of our findings based on Erb1_416-807_ structure from yeast. As shown in the Fig 8a the propeller appeared in the eluate from poly(U) beads. To investigate whether the interaction occurred through a well-defined surface that could be saturated, we incubated the protein with free poly(U) before it was loaded on the beads. The amount of the protein that could stably bind the poly(U) beads decreased in presence of 0.1mg/ml and 1mg/ml free poly(U), indicating saturation of the binding site (Fig 8b). The binding affinity was good because it was still detectable even when heparin was added to the reaction mixture (Fig 8c). Fluorescence experiments were carried out in order to estimate the binding affinity. Emission spectrum of intact ChErb1_432-801_ shows a maximum wavelength at 340 nm (Fig 8d), indicating that the tryptophans in the structure are partially buried, as shown in the X-ray structure. Therefore, if the RNA binding occurred in the proximity of the indole moiety, we should expect changes in the fluorescence spectra upon addition of polyU, and therefore we could use these changes to measure the affinity constants by using Eq (1). We acquired spectra at growing concentrations of polyU (Fig 8e), and the changes in the fluorescence emission spectra at 315 nm allowed us to determine the constant (similar changes were observed at other wavelengths either by excitation at 280 or 295 nm). The apparent *K*_D_ of the complex was 3 ± 2 μM. The fact that an exposed tryptophan (Trp682, red oval in the Fig 7a) is at the vicinity of the positively charged stretch (likely to participate in RNA binding), provides a good indication that the interaction takes place through the proposed area and explains the change in fluorescence upon binding of the nucleic acid. We also calculated binding affinity between ChErb1_432-801_ and a 15 nucleotide-long biotinylated poly(U) using biolayer interferometry. The dissociation constant (K_D_) was 0.17 μM (Fig 8f).

## Discussion

Our initial studies on Erb1 show that it is an unstable protein that rapidly undergoes proteolytic degradation when overexpressed in *E*. *coli* even upon binding to its functional partner Nop7. Since the interaction of Nop7 and Erb1 is strong (they co-elute in size exclusion chromatography), the proteolytic cleavage occurs somewhere between Nop7 binding site and the ß-propeller domain. In fact, Bop1, mammalian ortholog of Erb1, was predicted to possess a PEST (proline, glutamic acid, serine and threonine rich sequence) element in its central region that overlaps with the predicted Nop7 binding sequence [2]. The SitePrediction tool [44] confirmed the presence of a conserved PEST sequence in Erb1 in the same position as for Bop1. PEST motifs are clusters of charged amino acids that form flexible and solvent exposed loops that are linked to protein destabilization and degradation in eukaryotic systems [45]. Erb1 protein used in our study was expressed in bacteria where no PEST-related protein degradation mechanism has been described but it is possible that, despite Nop7 binding, the region is easily accessible for *E*. *coli* proteases, leading to a rapid Erb1 cleavage. It is known that Nop7/Erb1 complex is only stable when bound to Ytm1 and the predicted Ytm1 binding site is located in proximity of the PEST sequence and the β-propeller domain [6]. The interdependent stability of these factors could mark one of the many control checkpoints during ribosome assembly, in which the proteasome pathway might be involved.

The structure of the carboxy-terminal domain of Erb1 from *Saccharomyces cerevisiae* reveals that it adopts the classical seven-bladed β-propeller conformation but, at the same time, it presents a series of features that makes it distinguishable from the rest of the WD repeat-containing proteins. The sequence of the propeller is well conserved within the members of Erb1/Bop1 family but is quite dissimilar to other proteins with the same domain resulting in another good example of how the propeller fold is maintained throughout the evolution in spite of poor sequence conservation.

Even though, there is no clear evidence for Erb1Ct function in the cell, it is likely to be involved in mediating protein-protein or protein-rRNA interactions in eukaryotic ribosome assembly. Search of possible binding interfaces showed that the β-propeller of Erb1 contains several regions that might bind various ligands. These patches are conserved and appear in areas that, in other WD40-containing proteins, were shown to participate in binding to different targets. Since the charge on the surface of the propeller suggests that it could bind RNA, we confirm this hypothesis in vitro proving that a good-affinity binding takes place between the C-terminal domain and polyuridylic acid. Although we could also observe interaction between the β-propeller and DNA (data not shown), from a functional point of view RNA binding seems more likely to occur in vivo. The specificity of the interaction might be provided by Nop7/Erb1 attachment site on the nascent ribosome which results in proximity of Erb1 C-term to rRNA. This binding could contribute to the stability of Nop7 sub-complex association with pre-ribosome. We identified a region close to the central channel opening, on the narrow side of the propeller, that includes few conserved residues that mediate protein-protein interactions and appear in the same position as in Lis1 protein, a platelet-activating factor that binds to dynein and other ligands through this conserved surface [41]. In addition, in several β-propellers the same region was shown to be responsible for binding to other proteins or ligands and it was proposed as one of the most common interaction sites within the domain [46].

**Fig 9:**
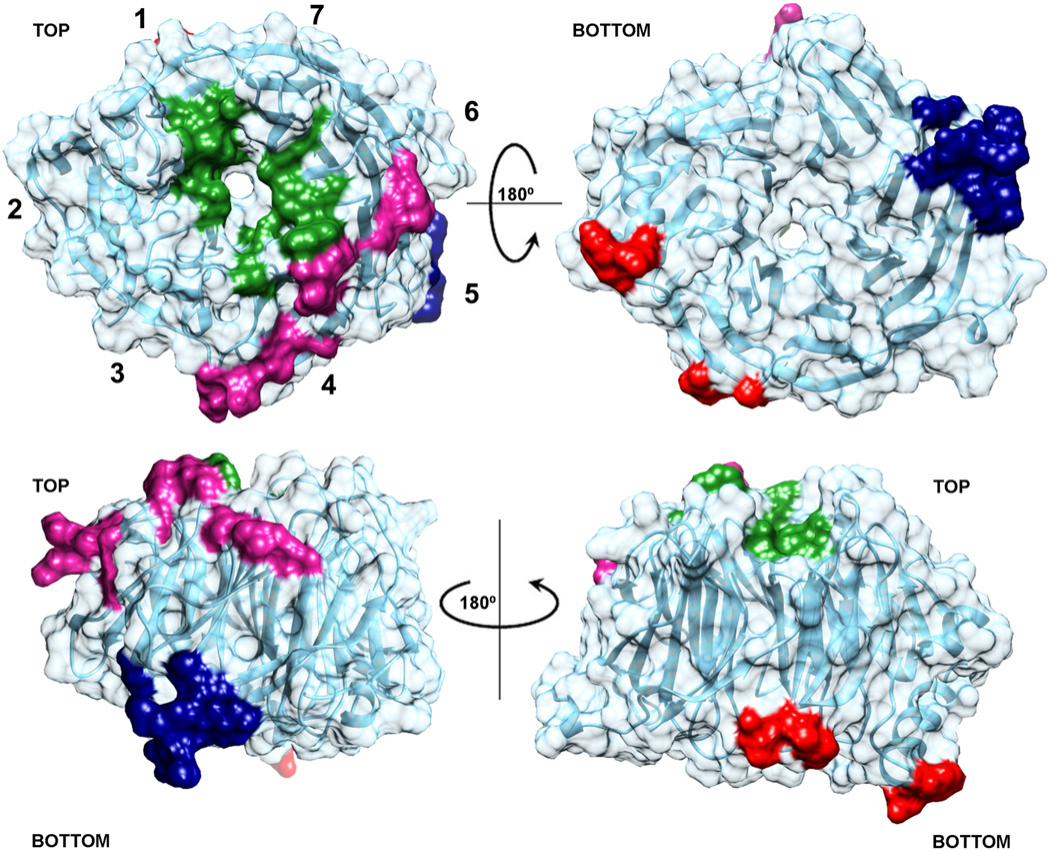
Summary of the areas of Erb1Ct that are more likely to mediate protein-protein or protein-RNA interactions. The region corresponding to the conserved hot-spot residues is shown in green, the positively charged stretch of amino acids that might interact with RNA is highlighted in magenta, navy blue and red areas correspond to the conserved parts of the domain that establish important crystal contacts with symmetrically related molecules.

Recently, it has been postulated that the WD40 proteins can also establish interactions by additional extensions or insertions that can occur between blades of the propeller [47]. It could be valid for Erb1 because, as we show here, it contains a long region that is introduced into WD2 and forms two visible α-helices and an additional strand. Residues in the propeller, outside the insertion, can contribute to the folding of the observed secondary motifs in this area. While the extra strand in blade 2 is probably necessary for structural stability of the inserted region and the propeller, the helices form an important protrusion that could serve as recognition motifs that selectively bind a ligand. In fact, the part that is not visible in the model (residues 535–570)—probably due to its flexibility and lack of secondary structure (as shown by the spectroscopic findings observed with the isolated region)—could become ordered and more rigid upon binding to a protein or nucleic acid. Since the top entrance of the tunnel and the helices from the insertions are located on opposite sides of the propeller, it is possible that the domain recruits two different binding partners at the same time (for a summary of the putative binding sites see Fig 9). It is also worth to note that C-D loop in blade 6 noticeably projects out of the bottom plane of the propeller in order to penetrate into a cavity of a symmetry-related molecule providing additional segments capable of interacting with other proteins. Moreover, if we take into consideration that Erb1 carries Nop7 and Ytm1 binding motifs towards its N-terminus it is plausible that the full length protein can establish multiple interactions within pre-ribosomal particles acting as a scaffold that plays an essential role in 60S maturation.

Taken together, our observations demonstrate that the C-terminal domain of Erb1 is likely to form part of a protein complex and is capable of binding RNA unspecifically through a defined surface. Although Granneman *et al*. showed that Erb1 bound to a specific region of 25S rRNA [9], they did not define the exact portion of the protein involved in the interaction. Our findings clearly suggest that the β-propeller domain might mediate this union. The size of the domain, its degree of conservation and the ability to bind nucleic acids supports the idea that Erb1 requires its carboxy-terminus to associate with other factors. Further functional studies need to be carried out in order to review the role of Erb1Ct in ribosome biogenesis especially if its propensity to bind RNA is considered.
