# Correction: The Carboxy-Terminal Domain of Erb1 Is a Seven-Bladed ß-Propeller that Binds RNA

**DOI:** 10.1371/journal.pone.0128164

**Published:** 2015-05-15

**Authors:** The PLOS ONE staff

The first author’s first and last name are switched. The correct name is Marcin Wegrecki. The correct citation should be:

Wegrecki M, Neira JL, Bravo J (2015) The Carboxy-Terminal Domain of Erb1 Is a Seven-Bladed ß-Propeller that Binds RNA. PLoS ONE 10(4): e0123463. doi:10.1371/journal.pone.0123463

The publisher apologizes for the error.
